# Identification of Isopeptides Between Human Tissue Transglutaminase and Wheat, Rye, and Barley Gluten Peptides

**DOI:** 10.1038/s41598-020-64143-9

**Published:** 2020-05-04

**Authors:** Barbara Lexhaller, Christina Ludwig, Katharina Anne Scherf

**Affiliations:** 1 Leibniz-Institute for Food Systems Biology at the Technical University of Munich, Lise-Meitner-Str. 34, 85354 Freising, Germany; 2 Bavarian Center for Biomolecular Mass Spectrometry (BayBioMS), Technical University of Munich, Gregor-Mendel-Str. 4, 85354 Freising, Germany; 3 Department of Bioactive and Functional Food Chemistry, Institute of Applied Biosciences, Karlsruhe Institute of Technology (KIT), Adenauerring 20a, 76131 Karlsruhe, Germany

**Keywords:** Proteomics, Coeliac disease

## Abstract

Celiac disease (CD) is a chronic immune-mediated enteropathy of the small intestine, which is triggered by the ingestion of storage proteins (gluten) from wheat, rye, and barley in genetically predisposed individuals. Human tissue transglutaminase (TG2) plays a central role in the pathogenesis of CD, because it is responsible for specific gluten peptide deamidation and covalent crosslinking, resulting in the formation of N^ε^-(γ-glutamyl)-lysine isopeptide bonds. The resulting TG2-gluten peptide complexes are assumed to cause the secretion of anti-TG2 autoantibodies, but the underlying mechanisms are only partly known. To gain more insight into the structures of these complexes, the aim of our study was to identify TG2-gluten isopeptides. With the use of discovery-driven as well as targeted nanoscale liquid chromatography tandem mass spectrometry, we detected 29 TG2-gluten isopeptides in total, involving seven selected TG2 lysine residues (K205, K265, K429, K468, K590, K600, K677). Several gluten peptides carried known B-cell epitopes and/or T-cell epitopes, either intact 9-mer core regions or partial sequences, as well as sequences bearing striking similarities to already known epitopes. These novel insights into the molecular structures of TG2-gluten peptide complexes may help clarify their physiological relevance in the initiation of CD autoimmunity and the role of anti-TG2 autoantibodies.

## Introduction

Celiac disease (CD) is defined as a chronic immune-mediated inflammatory disorder of the small intestine initiated by the storage proteins (gluten) of wheat, rye and barley in genetically predisposed subjects^1^. The ingestion of gluten causes villous atrophy, lymphocyte infiltration and the stimulation of CD4^+^ T cells against gluten epitopes in CD patients. These epitopes are presented by the human leukocyte antigen (HLA) class II alleles HLA-DQ2.5, HLA-DQ2.2 and HLA-DQ8 of the major histocompatibility complex (MHC) expressed on B cells and antigen-presenting cells. The presentation of gluten peptides leads to the activation of CD4^+^ T cells, which are the main effector cells for immunologic processes^2,3^.

Human tissue transglutaminase (TG2), a Ca^2+^-dependent protein-glutamine γ-glutamyltransferase (EC 2.3.2.13), is ubiquitously expressed and catalyses the deamidation of glutamine residues or the crosslinking reaction (transamidation) between a glutamine and a lysine residue to form a covalent N^ε^-(γ-glutamyl)-lysine isopeptide bond^4^. The TG2-mediated deamidation converts certain glutamine residues to glutamic acid residues by releasing ammonia and incorporating water. This leads to an introduction of negative charges in gluten peptides following a distinct pattern, e.g., the glutamine residues in the sequences QXP, QXXF(Y/W/M/L/I/V) or QXPF(Y/W/M/L/I/V), where X designates any other amino acid except P, are preferentially targeted^5^. This introduction of negatively charged amino acids increases the binding affinity of gluten peptides to the HLA molecules and enhances their antigenicity in CD patients^6^. During transamidation, the γ-carboxamide group of a protein-bound glutamine serves as acyl donor that is transferred to an acyl acceptor, such as small, biogenic amines or an ε-amino group of protein-bound lysine to form a crosslink^7,8^. The modification of gluten peptides by TG2 is known as a critical event in the pathomechanism of CD, particularly as TG2-gluten peptide complexes are formed^8^. Patients with active CD have specific anti-TG2 IgA (and IgG or IgM) antibodies^9^ and the formation of these antibodies is dependent on the ingestion of gluten. In previous studies, TG2 was identified as the predominant autoantigen of CD^10^. At the moment, there are different models to explain the formation of autoantibodies against TG2. It has been assumed that gluten-specific CD4^+^ T cells presented in the context of HLA-DQ2.5 or -DQ8 provide help to TG2-specific B cells^11^. After this initiation, different ways for gluten uptake by B cells and the role of B cell receptors (BCR) are possible: (i) BCR take up TG2-gluten peptide complexes and present them to gluten-specific CD4^+^ T cells, which provide help to B cells for the formation of anti-TG2 antibodies (original hapten-carrier-model^12^). In addition, (ii) the BCR may be crosslinked to neighboring BCRs by TG2 and this process contributes to B-cell activation. Evidence for these models comes from previous studies that TG2 can crosslink TG2 molecules into multimeric complexes, which can additionally incorporate gluten peptides. These multimers stimulate TG2-specific B cells and are presented to gluten-specific T cells^13^. (iii) The BCR might be crosslinked to gluten peptides through TG2 activity and thus be directly involved in uptake and presentation either in a single TG2-BCR complex or (iv) with a neighboring BCR^14^. After endocytosis of the BCR-gluten peptide complexes and TG2 by the receptor, the isopeptide bond between gluten peptide and BCR may again be hydrolyzed by TG2. This step releases the deamidated gluten peptide that will be subsequently linked to HLA-DQ and presented to CD4^+^ T cells.

In-depth studies about the formation of covalent TG2-gluten peptide complexes showed that six lysine residues of TG2 were involved in crosslinking with two different gluten peptides^8^ or even with TG2 molecules to create covalent TG2-TG2-multimers^13^. In addition, a reciprocal proteomics strategy using an α-gliadin-derived model peptide recently allowed the identification of 34 isopeptides involving 20 different lysine residues of TG2^15^. It is also known that, when confronted with a complex gluten peptide mixture, TG2 preferentially crosslinks peptides containing known CD-active T-cell epitopes to an acyl acceptor substrate such as 5-biotinamido-pentylamine^16^. However, there is no information as to which gluten peptides are good substrates for crosslinking to TG2, because all studies so far have worked only with selected gluten-derived model peptides and not with physiologically relevant enzymatic gluten hydrolysates due to their extreme heterogeneity^8,15^.

Therefore, the aim of our study was to apply our recently developed reciprocal mass spectrometric approach, including discovery-driven mass spectrometry^15^ and additional targeted proteomics, to complex gluten hydrolysates that had been incubated with TG2 and identify TG2-gluten isopeptides. We used well-characterized gluten protein types (GPTs) of wheat, rye and barley^17^ and extended our analysis strategy with additional confirmation of isopeptide identities by parallel reaction monitoring (PRM) LC-MS/MS as follow-up measurements.

## Results

### Experimental approach to identify TG2-gluten isopeptides

**Figure 1:**
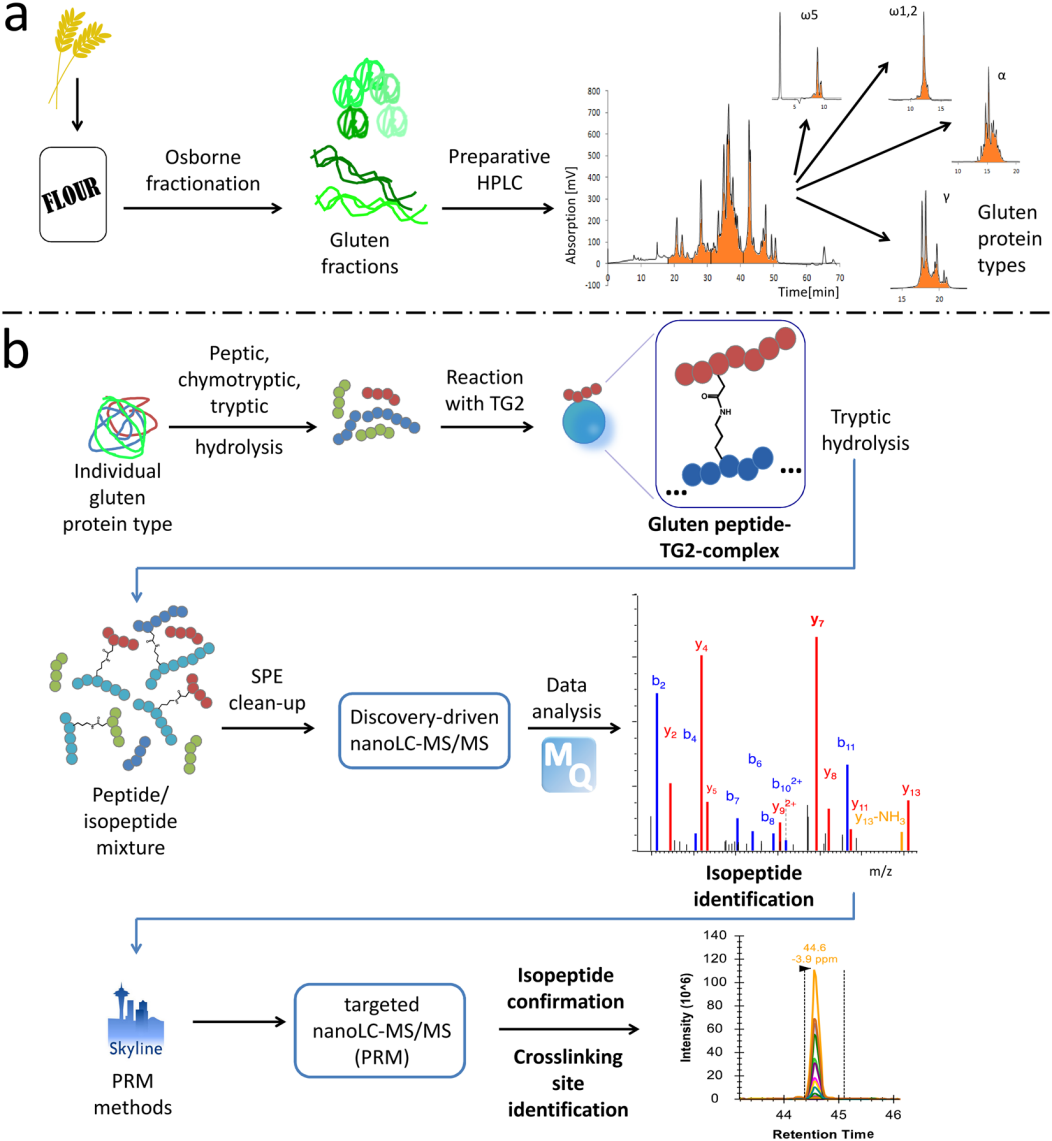
Workflow to identify isopeptides between gluten protein types of wheat, rye and barley and human TG2. (**a**) Extraction and separation procedure to obtain gluten protein types from wheat, rye and barley flours, respectively, (**b**) Proteomics workflow combining a reciprocal search strategy to identify isopeptides using discovery-driven mass spectrometry, MaxQuant, Skyline and parallel reaction monitoring (PRM). SPE: solid phase extraction; TG2: recombinant human tissue transglutaminase.

To reduce complexity compared to a total gluten hydrolysate, our experimental approach to identify TG2-gluten isopeptides started with the preparation of the following GPTs: α-gliadins, γ-gliadins, ω5-gliadins, ω1,2-gliadins, high- (HMW-GS) and low-molecular-weight glutenin subunits (LMW-GS) of wheat, ω-secalins, HMW-secalins, γ-75k-secalins and γ-40k-secalins of rye and C-hordeins, γ-hordeins, B-hordeins and D-hordeins of barley (Fig. 1a)^17,18^. The individual GPTs were hydrolysed using a combination of pepsin and chymotrypsin/trypsin to mimic the main enzymatic processes during gastrointestinal digestion^16,19^. Then, the resulting GPT hydrolysates were incubated with TG2, leading to the formation of TG2-gluten peptide complexes. These complexes were hydrolysed with trypsin followed by solid phase extraction (SPE) for clean-up of the isopeptide/peptide mixture and subsequent discovery-driven nanoscale liquid chromatography tandem mass spectrometry (nLC-MS/MS) analysis (Fig. 1b)^15^. The GPT blank controls without addition of TG2 were used to create customized protein databases (Table S1) for each GPT that were applied in the proteomics software MaxQuant (MQ)^20^. In order to identify TG2-gluten isopeptides, MQ searches for gluten peptides (α-side of the isopeptide) were performed against the appropriate GPT-database with each of seven selected TG2-peptides (β-side of the isopeptide) as modifications. These seven peptides (FL**K**NAGR, W**K**NHGCQR, IST**K**SVGR, LAE**K**EETGMAMR, DLYLENPEI**K**IR, Q**K**R, AV**K**GFR, lysine residue involved in crosslink formation highlighted in bold) containing the lysine residues K205, K265, K429, K468, K590, K600 and K677 from the TG2 amino acid sequence were selected as possible crosslinking sites. The lysine residues K590, K600 and K677 had previously been identified by Fleckenstein *et al*.^8^ and the lysine residues K205, K265, K429 and K468 additionally by Lexhaller *et al*.^15^. K590, K600 and K677 were known as preferred TG2 crosslinking sites also for TG2 self-multimerization^13^, while K205, K265, K429 and K468 were involved in the formation of isopeptides with high identification scores^15^. The tryptic TG2 peptides were chosen to contain only one lysine residue to reduce potential variability on the TG2-side. The identities of the isopeptides were confirmed by annotating the b- and y-fragments as well as internal fragment ions (double fragmentation on both crosslinked peptide sequences) calculated with the MS-Product feature of ProteinProspector^21^. The identities of the isopeptides as well as the crosslinking site localisations were verified by re-analysing all samples using targeted parallel reaction monitoring (PRM) nLC-MS/MS. Data analysis was performed with Skyline^22^ and additional manual curation. PRM analysis yields higher ion intensities, because it focuses on monitoring the predefined transitions from precursor to fragment ions. This higher overall intensity provided more fragments, especially around the crosslinking sites.

### Identification of isopeptides in wheat GPTs

**Figure 2:**
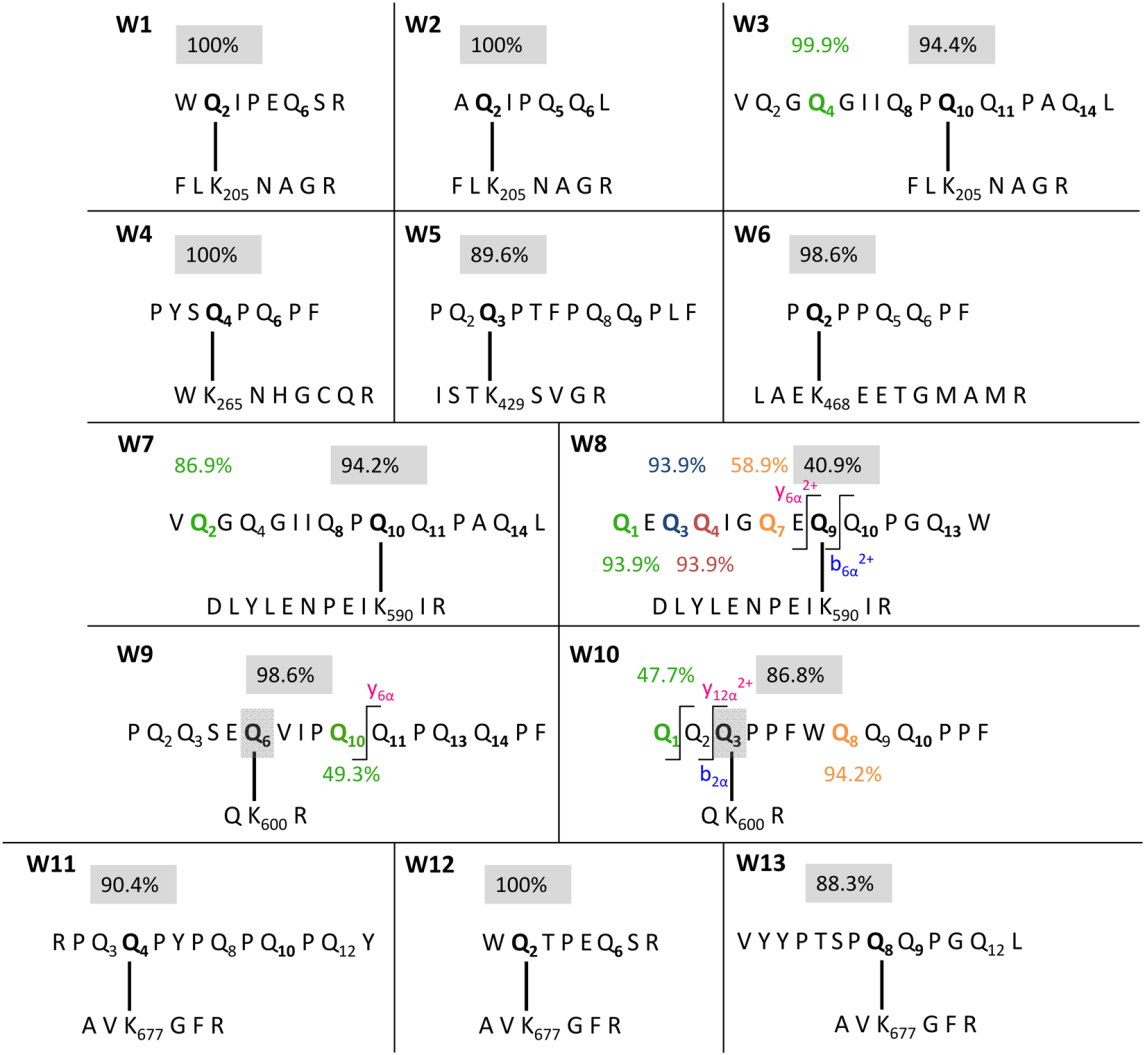
Schematic illustration of isopeptides between TG2 and wheat gluten proteins. (W1)–(W7) and (W11)–(W13), Isopeptides with localization probabilities >75%. (W8)–(W10), Isopeptides with crosslinking sites additionally confirmed by parallel reaction monitoring. The binding glutamine residues are given in bold, the binding probabilities for the crosslinks (MaxQuant) in the grey box and the deamidation probabilities for the glutamine residues in colours. Specific fragments used to confirm the binding sites are given in blue (b-fragments) and pink (y-fragments).

Altogether, 13 isopeptides were identified in the wheat GPTs. Table 1 shows the identified isopeptides (sorted by TG2-modification site) in each GPT, the gluten protein corresponding to the identified gluten peptide with UniProtKB accession number, name and organism, the MQ identification score, as well as the numbers of characteristic fragments identified in discovery-driven nLC-MS/MS experiments and of those that were confirmed using PRM. The γ-gliadin-GPT hydrolysate contained five isopeptides (W2, W3, W6, W7, W9) with four different TG2-crosslinking sites. Four isopeptides with three different TG2 peptides were identified in the α-gliadin-GPT hydrolysate (W1, W8, W11, W12), two isopeptides with two different TG2 peptides in the LMW-GS-GPT hydrolysate (W4, W10) and one isopeptide each in the HMW-GS-GPT hydrolysate (W13) and the ω1,2-gliadin-GPT hydrolysate (W5). No isopeptides were identified in the hydrolysate of ω5-gliadin-GPT. The structures of the isopeptides as well as the localization probabilities for the crosslinks and the deamidation are shown in Fig. 2.

**Table 1:** Isopeptides between TG2 and peptides derived from wheat gluten protein types.

Abb.^a^	TG2 lysine^b^	Gluten peptide^c^	GPT^d^	UniProtKB accession	UniProtKB name	Organism	MQ score^e^	Fragments (discovery)^f^		Manually checked (discovery)^g^		Fragments (targeted)^h^	
								α ^i^	β^j^	α	β	α	β
W1	K205	W**Q**IPEQSR	α	P04726	alpha/beta-gliadin clone PW1215	*T. aestivum*	49.37	10	4	18	13	9	—
W2	K205	A**Q**IPQQL	γ	A0A290XYW2	gamma-gliadin	*T. aestivum*	66.99	9	4	22	6	8	4
W3	K205	VQGQGIIQP**Q**QPAQL	γ	P08453	gamma-gliadin	*T. aestivum*	91.31	24	7	67	8	15	7
W4	K265	PYS**Q**PQPF	LMW	X2KVH9	alpha-gliadin	*T. aestivum*	59.21	9	1	16	2	7	—
W5	K429	PQ**Q**TFPQQPLF	ω1,2	R9XUE1	LMW-GS	*T. aestivum*	84.57	14	1	34	1	11	—
W6	K468	P**Q**PPQQPF^k^	γ	A0A290XYS8	omega-gliadin	*T. aestivum*	75.31	11	2	24	14	9	—
W7	K590	VQGQGIIQP**Q**QPAQL	γ	P08453	gamma-gliadin	*T. aestivum*	61.21	19	8	37	28	11	8
W8^l^	K590	QEQQIGQE**Q**QPGQW	α	B2LS24	HMW glutenin subunit type-2	*T. timopheevii*	55.26	12	2	28	—	14	2
W9^l^	K600	PQQSE**Q**VIPQQPQQPF	γ	A0A3B6UD61	uncharacterized protein	*T. aestivum*	104.05	17	—	39	—	14	—
W10^l^	K600	QQ**Q**PPFWQQQPPF	LMW	I3QPH0	low molecular weight glutenin subunit t128	*T. aestivum*	70.94	14	—	35	—	19	—
W11	K677	RP**Q**QPYPQPQPQY	α	A0A023WGB8	alpha-gliadin	*T. aestivum*	63.73	13	1	38	4	7	1
W12	K677	W**Q**TPEQSR	α	I0IT59	alpha/beta-gliadin	*T. aestivum*	64.42	13	3	13	3	19	3
W13	K677	VYYPTSP**Q**QPGQL	HMW	A0A1G4P1W4	HMW glutenin x-type subunit 1Bx6	*T. aestivum*	69.45	16	2	43	9	13	1

^a^Abb., abbreviation, ^b^Lysine residue in the TG2 sequence, K205: peptide FLKNAGR, K265: peptide WKNHGCQR, K429: ISTKSVGR, K468: LAEKEETGMAMR, K590: DLYLENPEIKIR, K600: QKR, K677: AVKGFR, ^c^Glutamine residues involved in crosslinking to TG2 are highlighted in bold, deamidation sites underlined, ^d^GPT, gluten protein type, α, α-gliadins, γ, γ-gliadins, ω1,2, ω1,2-gliadins, HMW, high-molecular-weight glutenin subunits, LMW, low-molecular-weight glutenin subunits, ^e^MQ, MaxQuant, ^f^Number of fragments identified by discovery-driven nLC-MS/MS and MaxQuant data analysis, ^g^Number of fragments identified by discovery-driven nLC-MS/MS and manual inspection of full scan spectra considering additional internal fragments calculated by ProteinProspector, ^h^Number of fragments identified by PRM analysis, ^i^α, α-side of the isopeptide (gluten peptide), ^j^β, β-side of the isopeptide (TG2 peptide), ^k^Unspecific cleavage at the C-terminal end (IP), ^l^Crosslinking site identified by PRM analysis.

**Figure 3:**
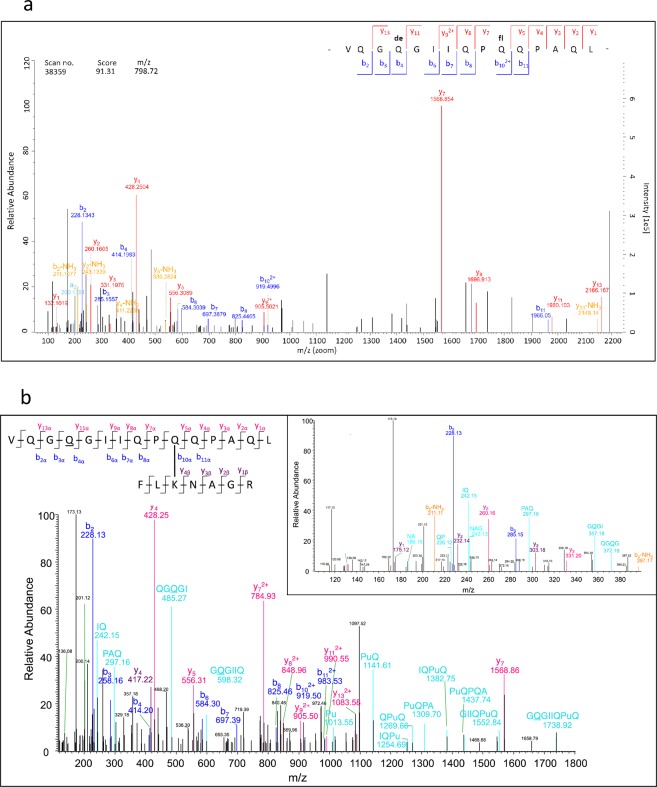
MS/MS spectrum of the isopeptide between VQGQGIIQPQQPAQL (γ-gliadin) and FLKNAGR (TG2). (**a**) Spectrum of the isopeptide annotated with fragments of the γ-gliadin peptide with TG2-peptide as modification as annotated by MQ Viewer (spectrum is shown decharged with fragments only single charged). The fragments are marked in different colours as follows: y-fragments in red, b-fragments in blue, a- and c-fragments in turquoise, fragments with losses of NH_3_ or CO marked in orange. (**b**) Spectrum of the isopeptide annotated manually with fragments of both sides of the isopeptides, calculated with ProteinProspector. The insert amplifies the range between *m/z* 100 to 400. The fragments are marked in different colours as follows: y-fragments of the γ-gliadin peptide in pink, b-fragments of the γ-gliadin peptide in blue, y-fragments of TG2 peptide in violet, a- and internal fragments in turquoise, fragments with losses of NH_3_ or CO marked in orange.

As an example, a very high MQ score (91.31) was obtained for the isopeptide VQGQGIIQP**Q**QPAQL/FL**K**NAGR (W3, Q and K involved in the isopeptide bond highlighted in bold, deamidation site underlined) based on the identification of 24 b- and y-fragments of the α-side (some fragments were identified without or with water- and ammonia-loss). First, the MQ search result of VQGQGIIQP**Q**QPAQL carrying the TG2 isopeptide modification “**fl**” (= FL**K**NAGR) at Q_10_ and a deamidation “**de**” at Q_4_ was loaded into MQ Viewer to have all b- and y-fragments annotated. These fragments were by default decharged by MQ Viewer to show them as single charged fragments (Fig. 3a)^23^. Additionally, in Fig. 3b, the annotation was done manually in the MS/MS spectrum by combining the information from the spectral annotation of MQ Viewer and 35 internal fragments calculated by ProteinProspector for confident localization of the deamidation and crosslinking sites in the isopeptides. The correct detection of isopeptide W3 was confirmed by targeted MS analysis using PRM^24,25^. The PRM data revealed high quality chromatographic peaks for 15 characteristic fragment ions, including b_6α_^+^ to b_8α_^+^ as consecutive series^26^ within the α-side, and seven fragments for the β-side modified at K with the deamidated VQGQGIIQPQQPAQL peptide. Q_10_ was identified as the crosslinking site with a localization probability of 94.4% and the deamidation at Q_4_ was detected with a probability of 99.9%.

The isopeptides W1-W7 and W11-W13 were already identified unambiguously by discovery-driven nLC-MS/MS and application of the confirmation parameters (at least seven identified b- or y-fragments, at least three fragments in a consecutive series and a crosslink localization probability ≥75%^15^). The additional PRM analysis confirmed these 10 identified isopeptides and their crosslinking and deamidation sites. However, the PRM data was essential to unambiguously localize the crosslinking site or some deamidation sites for the three isopeptides W8-W10. For this purpose, specific transitions around these sites were used to confirm the localization of the crosslinking or deamidation sites as shown in Fig. 2.

### Identification of isopeptides in rye GPTs

**Figure 4:**
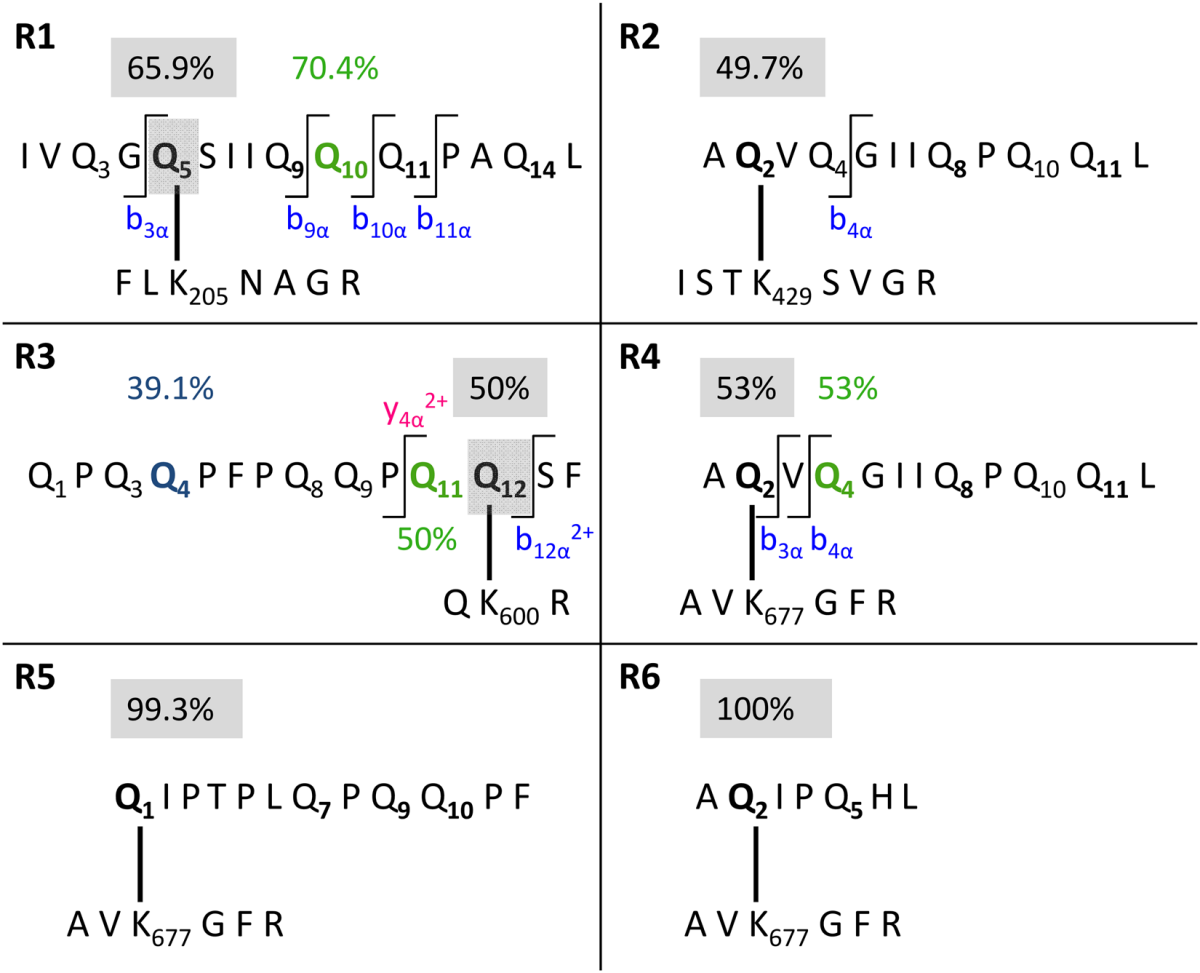
Schematic illustration of isopeptides between TG2 and rye gluten proteins. (R5)-(R6), Isopeptides with localization probabilities >75%. (R1)-(R4), Isopeptides with crosslinking sites additionally confirmed by parallel reaction monitoring. The binding glutamine residues are given in bold, the binding probabilities for the crosslinks (MaxQuant) in the grey box and the deamidation probabilities for the glutamine residues in colours. Specific fragments used to confirm the binding sites are given in blue (b-fragments) and pink (y-fragments).

Overall, six isopeptides were identified in the GPTs of rye (ω-secalins, HMW-secalins, γ-75k-secalins and γ-40k-secalins) (Table 2). Three isopeptides (R2-R4) crosslinked with three different TG2 peptides were detected in the γ-75k-secalin-GPT hydrolysate (Fig. 4). In the hydrolysate of the γ-40k-secalin-GPT, two isopeptides (R1, R6) with two different TG2 peptides were identified. One isopeptide (R5) with a gluten peptide derived from barley C-hordeins was identified in the ω-secalin-GPT hydrolysate, most likely due to high sequence homologies between rye ω-secalins and barley C-hordeins. No isopeptides were identified in the HMW-secalin-GPT hydrolysate.

**Table 2:** Isopeptides between TG2 and peptides derived from rye gluten protein types.

Abb.^a^	TG2 lysine^b^	Gluten peptide^c^	GPT^d^	UniProtKB accession	UniProtKB name	Organism	MQ score^e^	Fragments (discovery)^f^		Manually checked (discovery)^g^		Fragments (targeted)^h^	
								α ^i^	β^j^	α	β	α	β
R1^l^	K205	IVQG**Q**SIIQQQPAQL	γ40k	H8Y0N7	gamma prolamin	*S. cereale* ssp. *afghanicum*	68.97	17	4	34	4	13	3
R2^l^	K429	A**Q**VQGIIQPQQL	γ75k	A4GU91	75k gamma secalin	*S. sylvestre*	59.25	9	—	10	3	12	—
R3^l^	K600	QPQQPFPQQPQ**Q**SF	γ75k	H8Y0K1	gamma prolamin	*P. juncea*	80.75	14	—	39	2	10	—
R4^l^	K677	A**Q**VQGIIQPQQL	γ75k	A4GU91	75k gamma secalin	*S. sylvestre*	90.05	16	2	36	5	19	2
R5	K677	**Q**IPTPLQPQQPF	ω	Q41210	C-hordein	*H. vulgare*	57.18	14	1	41	3	10	1
R6	K677	A**Q**IPQHL	γ40k	H8Y0N7	gamma prolamin	*S. cereale* ssp. *afghanicum*	62.98	10	3	24	8	9	3

^a^Abb., abbreviation, ^b^Lysine residue in the TG2 sequence, K205: peptide FLKNAGR, K429: ISTKSVGR, K600: QKR, K677: AVKGFR, ^c^Glutamine residues involved in crosslinking to TG2 are highlighted in bold, deamidation sites underlined, ^d^GPT, gluten protein type, γ40k, γ-40k-secalins, γ75k, γ-75k-secalins, ω, ω-secalins, ^e^MQ, MaxQuant, ^f^Number of fragments identified by discovery-driven nLC-MS/MS and MaxQuant data analysis, ^g^Number of fragments identified by discovery-driven nLC-MS/MS and manual inspection of full scan spectra considering additional internal fragments calculated by ProteinProspector, ^h^Number of fragments identified by PRM analysis, ^i^α, α-side of the isopeptide (gluten peptide), ^j^β, β-side of the isopeptide (TG2 peptide), ^l^Crosslinking site identified by PRM analysis.

The isopeptides R5 and R6 were already identified unambiguously by discovery-driven nLC-MS/MS, because they fulfilled the confirmation parameters and the crosslinking sites were identified with localization probabilities of 99.3% and 100%, respectively. The PRM data from these isopeptides were used as confirmation. To identify the crosslinking sites in the other rye isopeptides (R1-R4), the identification and confirmation of specific fragments by PRM analysis was needed. Figure 4 shows the structures of these isopeptides as well as the MQ localization probabilities and the specific fragments used to confirm the crosslinking site.

### Identification of isopeptides in barley GPTs

**Figure 5:**
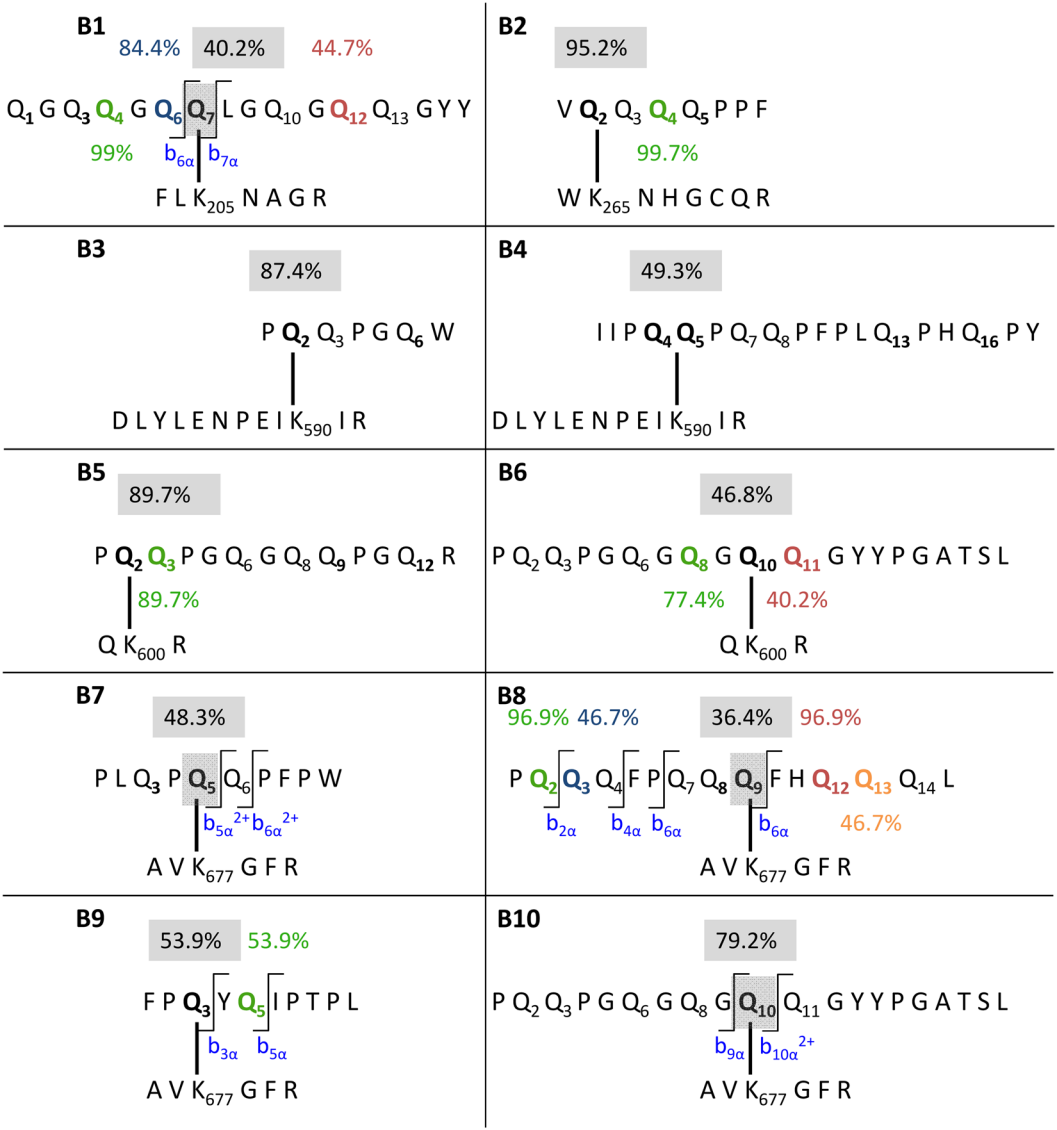
Schematic illustration of isopeptides between TG2 and barley gluten proteins. (B2), (B3) and (B5), Isopeptides with localization probabilities >75%. (B1), (B4) and (B6)–(B10), Isopeptides with crosslinking sites additionally confirmed by parallel reaction monitoring. The binding glutamine residues are given in bold, the binding probabilities for the crosslinks (MaxQuant) in the grey box and the deamidation probabilities for the glutamine residues in colours. Specific fragments used to confirm the binding sites are given in blue (b-fragments) and pink (y-fragments).

In total, ten isopeptides were identified in the GPTs of barley (C-hordeins, γ-hordeins, D-hordeins and B-hordeins) (Table 3). Five isopeptides (B1, B3, B5, B6, B10) with four different TG2 peptides were identified in the D-hordein-GPT hydrolysate and four isopeptides (B4, B7-B9) in the γ-hordein-GPT hydrolysate. The B-hordein-GPT hydrolysate contained one isopeptide (B2) with a gluten peptide derived from wheat LMW-GS, most likely again due to high sequence homologies between B-hordeins from barley and LMW-GS from wheat (Fig. 5). No isopeptides were detected in the hydrolysate of the C-hordein-GPT itself. However, one isopeptide identified within the ω-secalin-GPT was assigned to a C-hordein.

**Table 3:** Isopeptides between TG2 and peptides derived from barley gluten protein types.

Abb.^a^	TG2 lysine^b^	Gluten peptide^c^	GPT^d^	UniProtKB accession	UniProtKB name	Organism	MQ score^e^	Fragments (discovery)^f^		Manually checked (discovery)^g^		Fragments (targeted)^h^	
								α ^i^	β^j^	α	β	α	β
B1 ^l^	K205	QGQQGQ**Q**LGQGQQGYY	D	A0A2C9PIB7	high-molecular-weight glutenin subunit protein	*Ae. umbellulata*	59.90	13	—	25	4	16	—
B2	K265	V**Q**QQQPPF	B	V9P6N2	LMW-i glutenin subunit 1	*T. aestivum*	85.85	8	2	9	1	8	2
B3	K590	P**Q**QPGQW	D	I6TRS8	D-hordein	*H. vulgare*	44.97	7	13	11	21	7	13
B4	K590	IIP**QQ**PQQPFPLQPHQPY^k^	γh	P17991	C-hordein	*H. vulgare*	44.20	10	7	17	10	11	7
B5	K600	PQ**Q**PGQGQQPGQR	D	I6TRS8	D-hordein	*H. vulgare*	121.20	19	—	31	2	14	—
B6	K600	PQQPGQGQG**QQ**GYYPGATSL^k^	D	I6TRS8	D-hordein	*H. vulgare*	82.36	18	—	35	—	24	—
B7^l^	K677	PLQP**Q**QPFPW	γh	Q41210	C-hordein	*H. vulgare*	72.55	9	1	23	3	8	1
B8	K677	PQQQFPQQ**Q**FHQQQL	γh	A0A0B5JD29	omega-gliadin	*T. aestivum*	52.73	12	—	36	4	16	1
B9^l^	K677	FP**Q**YQIPTPL	γh	Q40053	Hor1–17 C-hordein	*H. vulgare*	47.94	11	2	25	7	10	2
B10^l^	K677	PQQPGQGQG**Q**QGYYPGATSL	D	I6TRS8	D-hordein	*H. vulgare*	106.36	23	2	41	3	24	2

^a^Abb., abbreviation, ^b^Lysine residue in the TG2 sequence, K205: peptide FLKNAGR, K265: peptide WKNHGCQR, K590: DLYLENPEIKIR, K600: QKR, K677: AVKGFR, ^c^Glutamine residues involved in crosslinking to TG2 are highlighted in bold, deamidation sites underlined, ^d^GPT, gluten protein type, D, D-hordeins, B, B-hordeins, γh, γ-hordeins, ^e^MQ, MaxQuant, ^f^Number of fragments identified by discovery-driven nLC-MS/MS and MaxQuant data analysis, ^g^Number of fragments identified by discovery-driven nLC-MS/MS and manual inspection of full scan spectra considering additional internal fragments calculated by ProteinProspector, ^h^Number of fragments identified by PRM analysis, ^i^α, α-side of the isopeptide (gluten peptide), ^j^β, β-side of the isopeptide (TG2 peptide), ^k^Both crosslinking sites are possible and could not be identified unambiguously due to missing fragments, ^l^Crosslinking site identified by PRM analysis.

The isopeptides B2, B3 and B5 were already detected unambiguously by discovery-driven nLC-MS/MS experiments and the PRM analyses were only used for confirmation. The localization probabilities for the crosslinking sites were between 87.4% and 95.2% (Fig. 5). In comparison, PRM analyses were necessary to detect the specific fragments around the crosslinking sites in B1, B7, B9 and B10 and confirm the localization of the crosslinks (Fig. 5).

Regarding the isopeptide B4, the localization probability was 49.3% for the crosslink at Q_4_ or Q_5_, respectively. The PRM data also did not reveal the exact position of the crosslink, because the specific transitions for these two sites were not detectable. The isopeptide B6 was identified with two deamidation sites, one of which was detected clearly with a localization probability of 77.4% at Q_8_. The positions of the second deamidation and the crosslinking site were ambiguous with localization probabilities of 51.0% at Q_10_ or 40.2% at Q_11_ for the deamidation and 46.8% at Q_10_ or 39.8% at Q_11_ for the crosslink. Even the PRM experiments did not provide any further information, so that the deamidation and crosslinking sites could not be assigned unequivocally within B6.

In the isopeptide B8, the crosslinking site was identified at various positions with various low localization probabilities by discovery-driven nLC-MS/MS: Q_8_ with 27.3%, Q_9_ with 36.4% and Q_12_, Q_13_, and Q_14_ with 12.0%, respectively. The positions Q_2_ (localization probability: 96.9%) and Q_3_ (localization probability: 46.7%) of the two deamidated glutamine residues in the N-terminal part of the sequence were verified due to the specific transitions b_2α_^+^ to b_4α_^+^, and the position of the crosslinking site could be confirmed at Q_9_ based on the detection of the characteristic b_6α_^+^ to b_9α_^+^ fragments after PRM analysis. Q_12_ had a deamidation probability of 96.9%, so that only the exact positions of the fourth deamidation in the rear part (Q_13_ or Q_14_) could not be assigned unambiguously due to missing specific fragments.

## Discussion

In this study, we applied a reciprocal proteomics strategy, including discovery-driven^15^ as well as targeted MS measurements, to complex gluten hydrolysates and identified isopeptides between TG2 and gluten peptides. To get well-defined gluten raw materials, GPTs were isolated by modified Osborne fractionation following preparative RP-HPLC and characterized as described before^17,18^. In total, 13 isopeptides of wheat GPTs, six of rye GPTs and ten of barley GPTs were detected crosslinked to peptides containing any of the seven selected TG2-lysine residues (K205, K265, K429, K468, K590, K600, K677). The crosslinking sites were unambiguously identified by discovery-driven nLC-MS/MS with localization probabilities of >75% in 18 out of 29 isopeptides. The additional PRM analyses on the ambiguously identified crosslinks in 11 isopeptides were used to clearly assign the crosslinking site. This method enabled the identification of the exact crosslinking and deamidation sites in 8 of the remaining 11 isopeptides due to the detection of the characteristic fragments around the modified sites. Only one deamidation site (B8), one crosslinking site (B4) as well as one deamidation and one crosslinking site (B6) could not be assigned unambiguously. However, we were able to identify the subpart of the amino acid sequence, where the modified glutamines are located most likely.

No isopeptides were detected in the hydrolysates of ω5-gliadins, HMW-secalins and C-hordeins. This may have several causes, including poor digestibility of the proteins, especially for ω5-gliadins^27^, comparatively low percentages of the respective GPT within the isolate, especially for C-hordeins^17^, isopeptide concentrations that were below the limit of detection or even no formation of isopeptides. Due to the multitude of potential pairings considering that the TG2 sequence contains 32 lysine residues in total, we decided to focus our data evaluation on the seven selected TG2-lysine residues within the specific peptides that had been reported as reactive sites in previous investigations^8,15^. With the gluten peptide side also unknown prior to our investigation, including all 32 lysine residues would have dramatically increased the search space at the cost of decreasing confident isopeptide identification. However, our intentional limitation to these seven lysine residues also implies that we may have missed isopeptides, if they contained any other TG2-derived lysine peptide.

The gluten peptides involved in isopeptide formation were not always matched to the corresponding proteins that would be primarily expected in the respective GPT. Each isopeptide dataset was searched against the GPT-specific database that was generated during the discovery-driven experiment with the GPT blank controls. Nevertheless, these GPT-specific databases partly contained proteins from other closely related plant species due to incomplete or unannotated protein entries in the UniProtKB database^28^. In some cases, the gluten peptides were derived from a different *Triticum* species like *T. timopheevii* (W8) or from different *Secale* species including *Psathyrostachys juncea* (Russian wild rye) (R1-R4, R6). One peptide present in the rye ω-secalin hydrolysate was matched to a protein sequence from *H. vulgare* (R5) and, vice versa, two peptides from the barley γ- and B-hordein hydrolysates corresponded to protein sequences from *T. aestivum* (B2, B8). This can be explained with the close phylogenetic relationship of wheat, rye and barley that causes extensive amino acid sequence homologies, especially in the repetitive domains^29,30^. Several gluten peptides also contained missed peptic/tryptic/chymotryptic cleavages sites, as is known to occur frequently during gluten digestion^31,32^. To enhance the quality of correct protein identifications, it might be useful to search in other, curated databases, which include more complete gluten entries^33^.

The approach with TG2 and GPTs described here has to be seen as a two-component model system with simulated gastrointestinal digestion. The crosslinking reactions were performed using isolated fractions of wheat, rye and barley proteins and this is rather far away from the real conditions, where gluten proteins are part of a complex food matrix. The simulated digestion model is based on physiological conditions including the three gastrointestinal enzymes trypsin, pepsin and chymotrypsin, but without the action of other enzymes, e.g., brush-border enzymes. This design was chosen deliberately, because the additional action of several enzymes with different cleavage specificities would have made the MS data evaluation much more complicated and increased the peptide search space by several orders of magnitude. These limitations of the current study have to be considered carefully, because more gastrointestinal enzymes would produce more or maybe divergent peptides from a more complex matrix.

TG2 is known for its high reactivity with gluten peptides^34^, especially those harboring T-cell epitopes^16^. Depending on the neighboring C-terminal amino acids, TG2 specifically deamidates glutamine residues in the QXP-, QXXF(Y/W/M/L/I/V)- or QXPF(Y/W/M/L/I/V)-motifs (where X designates any amino acid except P) resulting in increased binding affinity of the gluten peptides to the CD-associated HLA molecules^35^. In contrast, the QXXP- or QP-motifs have been described as poor or no targets for TG2-mediated deamidation^5^. Thirteen of the 29 isopeptides carried a gluten peptide with at least one additional deamidation, of which five displayed the preferred QXP-motif (W9, R1, B2, B5, B9) and five the QXXF(Y/L/I)-motif (W3, R3, B1, B6, B8). Two gluten peptides were deamidated at the poor QXXP- (W10) and QP-motifs (R3), while the remaining deamidation sites were located in sequences with unknown effect on TG2-specificity. Non-enzymatic deamidation cannot be excluded in our experiments due to the slightly alkaline pH conditions during incubation with TG2 and tryptic digestion^36^, but our intent was to focus on the identification of crosslinking sites, rather than deamidation sites.

Among the 29 isopeptides, 12 crosslinking sites to TG2 were located in the preferred QXP-motif (W1–W3, W7, W8, W12, W13, R5, R6, B3, B5, B7) and three in the QXX(Y/I)-motif (R1, B9, B10). Five isopeptides had the crosslink within the QP-motif (W4–W6, W10, W11) and one within the QXXP-motif (W9) that are either no or poor targets for TG2. The other crosslinks were either not localized unambiguously (B4, B6) or involved QXXX-motifs (R2-R4, B1, B2, B8) that may or may not have an effect on TG2 specificity. In case of W4 no preferred target was available, but these results point to the fact that TG2 might not necessarily follow the known specificity when it comes to crosslinking TG2 molecules to gluten peptides instead of deamidation. However, further experiments would be necessary to study the mechanisms of crosslinking versus deamidation in more detail.

A further limitation of the current study is that it does not allow a differentiation if one TG2 molecule carries several gluten peptides crosslinked to different lysine residues or if there are several TG2 molecules that carry one gluten peptide each. In view of the relative distance between the active site of TG2 and the crosslinked lysine residues, it appears most likely that TG2 crosslinked the gluten peptides to other independent neighbouring TG2 molecules. In our well-defined model system, there were no other acyl acceptor substrates present except for TG2. However, this situation is uncommon under physiological conditions, where other extracellular matrix proteins such as collagen or fibronectin and free amines are always present^37,38^. To address this major limitation of the current study, further experiments would be necessary in the presence of other proteins or free amines as potential substrates for TG2.

Of the 26 different gluten peptides (the isopeptides W3/W7, R2/R4 and B6/B10 involve different TG2 lysine residues, but the same gluten peptide, respectively) identified as part of isopeptides, three contained three different complete 9-mer core regions of known T-cell epitopes^39^: IQPQQPAQL (DQ2.5.glia-γ2^40^) in W3 and W7, FRPQQPYPQ (DQ2.5-glia-α3^40^) in W11 (F at the N-terminal end missing due to chymotryptic cleavage) and QQPFPQQPQ (DQ2.5-glia-γ5^41^) in R3. The crosslinked glutamine residue in W3, W7 and W11 was located within the core region, whereas R3 had the crosslink after the core region, but within the truncated motif of the epitope DQ2.5-glia-γ1 (PQQSFPQQQ^42^) that contains a chymotryptic cleavage site. The DQ2.5-glia-α3 and DQ2.5.glia-γ2 epitopes had also been identified as preferred TG2 substrates by Dorum *et al*.^16^. Several of the other gluten peptides crosslinked to TG2 also show striking similarities with known T-cell epitopes. For example, B7 is identical to LQPQQPFPQ (DQ2.5-glia-γ4e^43^) except for the C-terminal W, while also being identical to PQPQQPFPW (DQ2.5-glia-ω2^44^) except for the N-terminal L. W9 and B4 contain seven and eight amino acids of QQPQQPFPQ (DQ2.5-glia-γ4c^41^), respectively. Multiple sequence alignment of all identified gluten peptides that were bound to TG2 revealed that the PQQP-motif was the most common feature in many gluten peptides. However, there were also variations such as PQQL and PQQS, while some peptides had a different sequence altogether (e.g., within B9 or B1) (Fig. S1). The alignment of the gluten peptides considering the deamidation sites essentially showed a similar picture (Fig. S2).

As the formation of stable gluten peptide-HLA complexes is the prerequisite for activating the gluten-reactive T-cell response^35^ these TG2-bound gluten peptides carrying known T-cell epitopes may contribute to enhanced T-cell reactivity. In turn, gluten-reactive T cells provide help to gluten-specific B cells with both receptor repertoires sharing a preference for deamidated gluten peptides with overlapping or adjacent recognition sequences^45,46^. Although eight of the gluten peptides we identified within the isopeptides were too short (only eight amino acids in five cases, or seven amino acids in three cases) to elicit binding to HLA-DQ2.5, -DQ2.2 or -DQ8 molecules, one (W1) did carry a sequence recognized by gluten-specific B-cells (IPEQ, WQIPEQ)^46^. Furthermore, the peptides W9, R3, R6, B4 and B7 contained the QPQQPF-motif^46^ and W11 the PXPQP-motif^45^, that are reported as important sequences for B-cell receptor recognition. Regarding TG2-specific B cells, the most likely route is that TG2-gluten peptide complexes are taken up through the B-cell receptor^12^, but our knowledge on the cooperation of gluten-reactive T cells and TG2-specific B cells in B-cell activation warrants further investigation^11^. Our findings on isopeptide formation between TG2 and gluten peptides from a complex gluten hydrolysate may help shed some more light into the complex interactions between HLA-DQ2/8 molecules, gluten-reactive T cells, gluten-specific B cells and TG2-specific B cells. The workflow combining discovery-driven and PRM nLC-MS/MS could also be adapted to other related questions, because TG2 is also known to interact not only with gluten, but also with extracellular matrix proteins, such as fibronectin^37,38^.

## Conclusion

We identified 29 isopeptides of TG2 with peptides from gluten hydrolysates from wheat, rye and barley *in vitro* using a reciprocal proteomics strategy. The model system does not rely on model peptides, but uses gluten proteins extracted from the flours and hydrolysed by three different gastrointestinal enzymes to mimic physiological conditions in a simplified form. In addition to discovery-driven mass spectrometry, all isopeptides were verified by targeted proteomics (PRM) that allowed the localization of the respective crosslinking site. These results provide novel insights into preferred TG2 substrates and the molecular structures of TG2-gluten peptide complexes. Several gluten peptides carried known B-cell and T-cell epitopes, either intact 9-mer core regions or partial sequences, as well as sequences bearing striking similarities to already known epitopes. Further research combining *in vitro* and *in vivo* experiments on the extent and the activation of B cells are needed to get more insights on the immunological and physiological relevance of these complexes. With the proteomics strategy in place, it would be interesting to gradually move away from the well-defined model system to studying TG2-mediated crosslinking under physiologically relevant conditions, e.g., with additional action of brushborder enzymes and in the presence of other acyl acceptor substrates such as other extracellular matrix proteins or free amines.

## Methods

### Material

All chemicals and solvents were at least HPLC or LC-MS grade. Recombinant human TG2 was purchased from Zedira (Darmstadt, Germany) as a purified and lyophilized protein produced in sf9 insect cells. Trypsin (from bovine pancreas, TPCK-treated, ≥10,000 BAEE U/mg protein), pepsin (from porcine gastric mucosa, 10 FIP U/mg) and α-chymotrypsin (from bovine pancreas, TLCK-treated, ≥40 U/mg of protein) were purchased from Sigma-Aldrich (Steinheim, Germany). The Retention Time Standardize Kit PROCAL (Proteome Tools Calibration Standard) was from JPT (Berlin, Germany).

### Grain Samples

Grains of wheat (cultivar (cv.) Akteur, harvest year 2011, I.G. Pflanzenzucht, Munich, Germany), rye (cv. Visello, harvest year 2013, KWS Lochow, Bergen, Germany), and barley (cv. Marthe, harvest year 2009, Nordsaat Saatzucht, Langenstein, Germany) were milled into white flour using a Quadrumat Junior Mill (Brabender, Duisburg, Germany) and sieved to a particle size of 200 μm. Then, the flours were allowed to rest for 2 weeks prior to the determination of moisture and protein contents (conversion factor N × 5.7) according to International Association for Cereal Science and Technology (ICC) Standards 110/1^47^ and 167^48^, respectively. The moisture contents were 14.59 ± 0.01% (wheat), 11.42 ± 0.01% (rye) and 12.09 ± 0.06% (barley) and the crude protein contents were 9.93 ± 0.14% (wheat), 5.81 ± 0.29% (rye) and 6.72 ± 0.04% (barley).

### Preparation of GPTs

The GPTs α-gliadins, γ-gliadins, ω5-gliadins, ω1,2-gliadins, HMW-GS and LMW-GS of wheat, ω-secalins, HMW-secalins, γ-75k-secalins and γ-40k-secalins of rye, and C-hordeins, γ-hordeins, D-hordeins and B-hordeins of barley were isolated as reported in detail by Schalk *et al*.^18^ and Lexhaller *et al*.^17^. Briefly, the protein fractions were isolated stepwise by modified Osborne fractionation from wheat, rye and barley flours using salt solution (0.4 mol/l NaCl with 0.067 mol/l Na_2_HPO_4_/KH_2_PO_4_, pH 7.6) to obtain the albumins/globulins, ethanol/water (60/40, v/v) to obtain the prolamins and glutelin extraction solution (2-propanol/water (50/50, v/v)/0.1 mol/l Tris-HCl, pH 7.5, containing 2 mol/l (w/v) urea and 0.06 mol/l (w/v) dithiothreitol (DTT)) at 60 °C under nitrogen to obtain the glutelins. The supernatants of each prolamin and glutelin fraction were combined, concentrated, lyophilized and re-dissolved for preparative RP-HPLC. After filtration of the prolamin and glutelin solutions (0.45 μm), the GPTs were separated on a Jasco HPLC (Jasco, Gross-Umstadt, Germany) according to their retention times, collected from several runs, pooled, lyophilized and stored at −20 °C until use. Then, the GPTs were characterized by RP-HPLC, SDS-PAGE and discovery-driven mass spectrometry to verify their identities and purities as already reported in detail^17,18^.

### Enzymatic digestion of GPTs

Each GPT was suspended in 0.02 mol/l HCl (pH 2) and hydrolyzed with pepsin at an enzyme:substrate ratio of 1:20 (w/w) for 60 min at 37 °C. After adjusting the pH to 6.5 with sodium phosphate buffer (50 mmol/l), trypsin and chymotrypsin were added at an enzyme:substrate ratio of 1:40 (w/w), respectively and hydrolyzed for 120 min at 37 °C^16,19^. The samples were heated for 10 min at 95 °C to stop proteolysis, centrifuged and filtered. For the following crosslinking reaction with TG2, the samples were dried using a vacuum centrifuge (37 °C, 4 h, 800 Pa), reconstituted in TRIS/HCl buffer (0.1 mol/l, pH 7.4, 10 mmol/l CaCl_2_) and the resulting peptide concentrations were estimated with a NanoDrop Micro-UV/VIS spectrophotometer and the protein A205 application (NanoDrop One, Thermo Scientific, Madison, USA) at 205 nm, which can be used to determine peptide concentrations based on the absorption of the peptide bonds.

### Crosslinking reaction of TG2 and GPT hydrolysates

The reaction of TG2 (0.16 nmol/l) with each GPT hydrolysate was performed in TRIS/HCl buffer (0.1 mol/l, pH 7.4, 10 mmol/l CaCl_2_) at a molar ratio of TG2:GPT hydrolysate of 1:150 at 37 °C for 120 min.^15^ To inactivate TG2, all samples were heated at 95 °C for 10 min. The negative controls were prepared by adding the GPT hydrolysates after inactivation of TG2. Additional GPT blank controls contained only GPT in TRIS/HCl buffer and were treated as described above just without TG2. The samples and the negative controls were prepared in triplicates; the GPT blank controls were also prepared in triplicates, but pooled prior to tryptic hydrolysis.

### Tryptic digestion and isopeptide clean-up

Enzymatic hydrolysis and peptide purification were carried out as described in detail by Lexhaller *et al*.^15^. Briefly, all samples, negative controls and GPT blank controls were hydrolyzed with trypsin at an enzyme:substrate ratio of 1:100 (w/w) at 37 °C for 24 h and the digestion was stopped with formic acid (FA, pH <2). Purification was done by solid phase extraction (SPE) using 50 mg Sep-Pak tC_18_ cc cartridges (Waters, Eschborn, Germany). After activation with methanol (1 ml), equilibration with acetonitrile/water/FA (80:20:0.1; 1 ml), and washing with acetonitrile/water/FA (2:98:0.1; 5 × 1 ml), the cartridges were loaded with the samples and washed again. The isopeptides and peptides were eluted with acetonitrile/water/FA (40:60:0.1; 2 ×0.5 ml), dried and reconstituted in FA (0.1%, v/v). Prior to nLC-MS/MS analysis, the peptide concentrations of the reconstituted samples were estimated again with the NanoDrop Micro-UV/VIS spectrophotometer at 205 nm. All samples were spiked with the PROCAL Mix (33 fmol/µl) and diluted in 96 well plates to a concentration of 200 ng/µl with acetonitrile/water/FA (2:98:0.1).

### Discovery-driven mass spectrometry

nLC-MS/MS analysis was performed on an Ultimate 3000 nanoHLPC system (Dionex, Idstein, Germany) coupled to a Q Exactive HF mass spectrometer (Thermo Fisher Scientific, Dreieich, Germany). The nanoscale LC system consisted of a trap column (75 µm × 2 cm, self-packed with Reprosil-Pur, C_18_, ODS-3, 5 µm resin, Dr. Maisch, Ammerbuch, Germany) and an analytical column (75 µm × 40 cm, self-packed with Reprosil-Gold, C_18_, 3 µm resin, Dr. Maisch). The injection volume was 2 µL (estimated peptide concentration: 0.16 µg/µL). The peptides were delivered to the trap column using solvent A0 (0.1% FA in water) at a flow rate of 5 µL/min and separated on the analytical column using a 60 min linear gradient from 4% to 32% solvent B at a flow rate of 300 nL/min (solvent A1, 5% DMSO, 0.1% FA in water; solvent B, 5% DMSO, 0.1% FA in acetonitrile)^49^. The MS was operated in data-dependent acquisition mode, automatically switching between MS1 and MS2 spectra to acquire full scans. The mass-to-charge (*m/z*) range for the acquisition of MS1 spectra was 360–1,300 *m/z* at an Orbitrap full MS scan (resolution: 60,000, automatic gain control (AGC) target value: 3e6, maximum injection time: 50 ms). In the MS2, the Top18 peptide precursors were automatically selected for fragmentation by higher energy collision-induced dissociation (isolation width: 1.7 Th, maximum injection time: 25 ms, AGC value: 1e5). Analysis was performed using 25% normalized collision energy at a resolution of 15,000.

### Preparation of GPT databases

Each GPT blank control was searched individually against a protein database containing all gliadin entries (January 2019; 5,958 entries), glutenin entries (January 2019; 4,488 entries), secalin entries (January 2019; 219 entries) and hordein entries (January 2019; 158 entries) of the UniProtKB database using MQ (software version 1.6.0.1)^20^. The parameters were set as follows: digestion mode: specific, enzyme: trypsin, pepsin, chymotrypsin, maximum missed cleavage sites: 2, variable modifications: deamidation (NQ), oxidation (M), main search peptide tolerance: 4.5 ppm, mass tolerance for fragment ions: 0.5 Da. All other parameters were used as default settings. All identified proteins in the proteinGroups.txt file were used to create an appropriate database for each GPT.

### Identification of TG2-gluten isopeptides

The Thermo Xcalibur full scan.raw files of each GPT (three samples and three negative controls) were directly used as input in MQ^20^ and searched against the appropriate GPT database. Seven peptides containing lysine residues (K205, K265, K429, K468, K590, K600, K677) from the TG2 sequence were selected as possible crosslinking sites in the isopeptides. The elemental compositions of these tryptic TG2 peptides were calculated *in silico* to configure the TG2-sides of the isopeptides as modifications in MQ. A formal subtraction of NH_3_ was necessary to use these peptides as modifications (TG2-modifications) in an isopeptide bond^15^. The parameters were set as follows for the individual search runs: digestion mode: specific, enzyme: trypsin, pepsin, chymotrypsin, maximum missed cleavage sites: 2, variable modifications: each TG2-modification in one single search run, deamidation (NQ), TG2-modifications: FL**K**NAGR, C_36_H_57_N_11_O_9_, W**K**NHGCQR, C_43_H_62_N_16_O_11_S, IST**K**SVGR, C_35_H_63_N_11_O_12_, LAE**K**EETGMAMR, C_55_H_93_N_15_O_20_S_2_, DLYLENPEI**K**IR, C_68_H_108_N_16_O_21_, Q**K**R, C_17_H_31_N_7_O_5_, AV**K**GFR, C_31_H_49_N_9_O_7_, max. number of modifications per peptide: 5, fasta files: appropriate GPT and TG2 (UniProtKB accession no. P21980) fasta files, minimum score for modified peptides: 40. All other parameters were used as default settings.

### Annotation of MS/MS fragments of the isopeptides

To confirm the identification and the respective crosslinking sites of the isopeptides, the b- and y-fragments of both sides were calculated with the MS-Product feature of the ProteinProspector webpage (v.5.22.1, University of California, San Francisco, CA, USA)^21^. The sequences of gluten peptides and the TG2-modifications were entered and the binding Q or K were replaced by “u” for the user-specified amino acid elemental composition of the other isopeptide site, respectively. ProteinProspector parameters were then set to calculate b-, y- and internal fragments and associated fragments due to water- and ammonia-loss. The charge states were calculated up to 5+ for the precursors and up to 3+ for the fragments.

### Isopeptide confirmation and creation of PRM methods

Skyline-daily (version 19.0.9.149)^22^ was used to confirm the identities of all detected isopeptides, to compare negative controls and samples and to create isolation lists for the PRM methods. To confirm the identified isopeptides and reject false positives, the sequences of the GPT-peptides were loaded into Skyline as the targets and modified with the appropriate TG2-modifications, a deamidation (−17 Da) or both, according to the MQ output. To identify the isopeptides from both sides, the reverse isopeptide sequence, i.e., the sequence of the TG2 peptide, was also loaded into Skyline and modified with the previously identified GPT peptide via a crosslink. Then, Skyline generated the appropriate precursors of all sequences. Every isopeptide was manually checked to fulfill the following parameters: (a) the retention time had to match with the identified retention time of the MQ search (ID), (b) comparison of retention time and isotopic dot product scores (idotp: generated from comparing the expected precursor isotopic distribution to the observed distribution; scored from 0–1 with 1 being the highest) among the triplicates using the graphical tools^22^, (c) reproducible detection of the isopeptide in the three replicates and absence in the negative controls; false positive matches in the negative controls were rejected, (d) the idotp had to be >0.9, (e) the threshold for unambiguous localization was set to a localization probability of >75% (MQ search). MS/MS libraries were built to generate the isolation lists for the isopeptides of each GPT. Therefore, the MQ output tables “msms.txt” of the searches of every modification were imported into Skyline. All identified isopeptides of one GPT and their reversed isopeptides with the appropriate GPT-modifications were summarized in one PRM method. This method was exported as an isolation list for use in the nLC-MS/MS system. A single isolation list and a single PRM method were created for each GPT.

### Targeted mass spectrometry

All PRM measurements were carried out using the exact same instrument and LC conditions as for the discovery-driven setup (see above). The MS was operated in unscheduled PRM mode with the following settings: MS1 resolution: 60,000, MS1 automatic gain control (AGC) target value: 3e6, MS1 maximum injection time: 100 ms, MS1 scan range 360–1300 *m/z*, quadrupole isolation window width: 1.7 Th, MS2 maximum injection time: 22 ms, MS2 AGC value: 1e6. High-energy collision-induced dissociation was performed using a normalized collision energy of 27.

### PRM data analysis

The Xcalibur.raw files of the PRM data were imported into Skyline separately for each GPT. The transitions of each target were checked manually and in comparison to the negative controls. To confirm the identified isopeptides and reject false positives, the following parameters were checked: (a) the retention time in the PRM data had to match with the identified retention time of the MQ search (ID) and the full scan data, (b) the comparison of retention time and idotp of the precursors among the triplicates had to fit using the graphical tools and no detection of the signals in the negative controls had to be observed, (c) according to Chen *et al*.^26^, at least seven identified b- or y-fragments had to match theoretical peptide fragments, (d) at least three fragments had to be consecutive in the peptide sequence. Every identified isopeptide was double-checked with the MQ search result in the MQ Viewer.

### Multiple sequence alignment of gluten peptides

All gluten peptides identified as part of the isopeptides were compiled into a peptide fasta file, either without or with deamidation at the sites we had detected. The multiple sequence alignment was done using MAFFT online version 7.452 on January 16, 2020 (Computational Biology Research Center, National Institute of Advanced Industrial Science and Technology, Tokyo, Japan) using the default settings and the L-INS-I algorithm^50^.

## Supplementary information

Supplementary information.

## Data Availability

The mass spectrometry proteomics data have been deposited to the ProteomeXchange Consortium (http://proteomecentral.proteomexchange.org) with the dataset identifier PXD017693 and are publicly available on Panorama Public (https://panoramaweb.org/8QUQ5F.url).
