# Supplementary material for: Identification of Isopeptides Between Human Tissue Transglutaminase and Wheat, Rye, and Barley Gluten Peptides
Source: Sci Rep. 2020 May 4;10:7426. doi: 10.1038/s41598-020-64143-9 (PMC7198585; doi:10.1038/s41598-020-64143-9)
Supplement: Supplementary file 1 — Supplementary information. [file 41598_2020_64143_MOESM1_ESM.pdf]

Identification of Isopeptides Between Human Tissue Transglutaminase and Wheat, Rye, and Barley Gluten Peptides

Supplementary Material

Barbara Lexhaller[1], Christina Ludwig[2], Katharina Anne Scherf[1,3]*

[1]Leibniz-Institute for Food Systems Biology at the Technical University of Munich, Lise-Meitner-Str. 34, 85354 Freising, Germany

[2]Bavarian Center for Biomolecular Mass Spectrometry (BayBioMS), Technical University of Munich, Gregor-Mendel-Str. 4, 85354 Freising, Germany

[3]Department of Bioactive and Functional Food Chemistry, Institute of Applied Biosciences, Karlsruhe Institute of Technology (KIT), Adenauerring 20a, 76131 Karlsruhe, Germany

Table S1. Number of identified proteins in each gluten protein type (GPT) blank sample.

GPTs wheat	Number of protein entries in database	GPTs rye	Number of protein entries in database	GPTs barley	Number of protein entries in database
HMW-GS	2233	HMW-secalins	586	D-hordeins	1673
ω1,2-gliadins	2470	ω-secalins	777	C-hordeins	1103
ω5-gliadins	1167				
LMW-GS	2225	γ-75k-secalins	807	B-hordeins	903
γ-gliadins	2270	γ-40k-secalins	1725	γ-hordeins	745
α-gliadins	2500				

```
W1                ----------WQIPEQSR---------------
W12               ----------WQTPEQSR---------------
W2                ----------AQIPQQL----------------
R6                ----------AQIPQHL----------------
B8                --------PQQQFPQQQFHQ--QQL--------
W5                -----PQQTF---PQQPLF--------------
W6                ----------PQPPQQPF---------------
W9                P--QQSEQVIPQQPQQPF---------------
B4                IIPQQPQQPFPLQPHQPY---------------
R3                ----QPQQPFPQQPQQSF---------------
B7                ----------PLQPQQPFPW-------------
R5                ------QIPTPLQPQQPF---------------
W8                ------QEQQIGQEQQPGQW-------------
B3                -------------PQQPGQW-------------
B5                -------------PQQPGQG--QQPGQR-----
B6                -------------PQQPGQGQGQQGYYPGATSL
W13               -------VYYPTSPQQPGQL-------------
W3                -----VQGQGIIQPQQPAQL-------------
R2                -----AQVQGIIQPQQL----------------
R1                I----VQGQSIIQ-QQPAQL-------------
W10               -------------QQQPPFWQ-QQPPF------
B2                -----------VQQQQPPF--------------
W4                ----------------PYSQ--PQPF-------
W11               ------------RPQQPYPQ--PQPQY------
B9                ----------FPQYQIPTPL-------------
B1                ---------QGQQGQQLGQG--QQGYY------
```

Figure S1. CLUSTAL format multiple sequence alignment of all non-deamidated gluten peptides involved in isopeptide formation with human transglutaminase 2 (MAFFT online version 7.452, Jan. 16, 2020).

```
W1              --------WQIPEQSR---------------
W12             --------WQTPEQSR---------------
W2              --------AQIPQQL----------------
R6              --------AQIPQHL----------------
B8              ------PEEQFPQQQFHEQQL----------
B8'             ------PEEQFPQQQFHQEQL----------
W3              ---VQGEGIIQPQQPAQL-------------
R2              ---AQVQGIIQPQQL----------------
R1              I--VQGQSIIQ-EQPAQL-------------
W5              -----------PQQTFPQQPL--F-------
R3              ----------QPQQPFPQQPEQSF-------
W6              --------PQPPQQPF---------------
B4              ------IIPQQPQQPFPLQPHQPY-------
W9              PQQSEQVIPEQPQQPF---------------
B7              --------PLQPQQPFPW-------------
R5              ----QIPTPLQPQQPF---------------
W8              ----EEEEIGEEQQPGQW-------------
B3              -----------PQQPGQW-------------
W13             -----VYYPTSPQQPGQL-------------
B6              -----------PQQPGQGEGQQGYYPGATSL
B5              -----------PEQPGQGQQPGQR-------
W10             ----------EQQPPFWEQQPP-F-------
B2              --------VQQEQPPF---------------
W4              --------------PYSQPQP--F-------
W11             ----------RPQQPYPQPQPQ-Y-------
B9              ----------FPQYEIPTPL-----------
B1              -------QGQEGEQLGQGEQG--YY------
```

Figure S2. CLUSTAL format multiple sequence alignment of all gluten peptides that are involved in isopeptide formation with human transglutaminase 2 considering the detected deamidation sites (MAFFT online version 7.452, Jan. 16, 2020).
